# Draft Genome Sequence of Comamonas jiangduensis Strain YW1^T^

**DOI:** 10.1128/MRA.00513-20

**Published:** 2021-02-11

**Authors:** Luis Johnson Kangale, Anthony Levasseur, Didier Raoult, Eric Ghigo, Pierre-Edouard Fournier

**Affiliations:** a Aix-Marseille University, IRD, AP-HM, SSA, VITROME, Marseille, France; b IHU-Méditerranée-Infection, Marseille, France; c Aix-Marseille University, IRD, AP-HM, MEPHI, Marseille, France; d Special Infectious Agents Unit, King Fahd Medical Research Center, King Abdulaziz University, Jeddah, Saudi Arabia; e Techno-Jouvence, Marseille, France; University of Maryland School of Medicine

## Abstract

Here, we report the draft genome sequence of Comamonas jiangduensis strain YW1^T^ (= DSM 100319^T^ = CSUR Q1714^T^ = CCTCC AB 2012033^T^  =  KACC 16697^T^). Comamonas jiangduensis is a new *Comamonas* species that was isolated from agricultural soil. The genome sequence from strain YW1^T^ has been assembled into 322 contigs for a total size of 2,758,586 bp with a G+C content of 59.1%.

## Abstract (precis)

Here, we report the draft genome sequence of Comamonas jiangduensis strain YW1^T^ (DSM 100319^T^, CSUR Q1714 ^T^, CCTCC AB 2012033^T^ ,  KACC 16697^T^). Comamonas jiangduensis is a new *Comamonas* species that was isolated from agricultural soil. The genome sequence from strain YW1^T^ has been assembled into 322 contigs for a total size of 2,758,586 bp, with a G+C content of 59.1%.

## ANNOUNCEMENT

The genus *Comamonas*, assigned to the family *Comamonadaceae* (*Betaproteobacteria*), was originally established by Davis and Park (1) in 1962. The *Comamonadaceae* family was validly published only in 1985, with Comamonas terrigena as the type species (2). Strain YW1^T^ was isolated from agricultural soil and was described as the type strain of Comamonas jiangduensis, which is a new biosurfactant-producing species (3). Currently, the complete genome sequence is not available; therefore, we describe the genomic features of C. jiangduensis strain YW1^T^. We acquired strain YW1^T^ (= DSM 100319) from the Deutsche Sammlung von Mikroorganismen und Zellkulturen GmbH (DSMZ), and we deposited it in the Collection de Souches de l’Unité des Rickettsies (CSUR) culture collection under the accession number Q1714. The bacterial strain was grown at 28°C for 24 h on Columbia blood agar (bioMérieux, Marcy l’Etoile, France). The bacterial genomic DNA was extracted using an EZ1 system and the DNA tissue kit (Qiagen, Hilden, Germany). The bacterial genomic DNA was quantified using a Qubit assay (Life Technologies, Carlsbad, CA, USA). To provide the full genome sequence of C. jiangduensis strain YW1^T^, we carried out two methods of sequencing. First, the bacterial genomic DNA was normalized at 0.2 ng/μl. Genomic DNA was next sequenced with MiSeq (4) technology (Illumina, Inc., San Diego, CA, USA) using the Nextera XT DNA sample preparation kit (Illumina) with the paired-end strategy. To prepare the paired-end library, dilution was performed to yield 1 ng of each genome as the input. Normalized libraries were pooled into a single library for sequencing on the MiSeq system. Automated cluster generation and paired-end sequencing with dual index reads were performed in a single 39-hour run (2 × 250 bp). Total information of 9.29 Gb was obtained from a cluster with a density of 1,012,000 clusters/mm^2^, with a cluster passing quality control filters of 92.90%. Within this run, the index representation for strain YW1^T^ was determined to 4.26% of index represention. The 19,461,691 paired-end reads of the MiSeq run were examined to evaluate the quality using FastQC v0.11.8 (5). Second, the bacterial genomic DNA was sequenced using a Nanopore strategy with a MinION sequencer (Oxford Nanopore Technologies) as described previously (6, 7). After a 2-hour run time and the end of the life of the flow cell, 1,140,000 reads were generated as raw data. Finally, sequencing reads were assembled using SPAdes (8) software (Galaxy 3.12.0+galaxy1) with default parameters. Contigs were combined using SSPACE v2.0 (9) and GapFiller v2.1.1 (10) with default parameters. Then, manual finishing was performed using sequence similarity searches and blocks conserved among the closest species, i.e., Comamonas terrigena strain NBRC 13299, Comamonas serinivorans strain DSM 26136, and Comamonas kerstersii strain 8943. Genomic annotation was performed using the NCBI Prokaryotic Genome Annotation Pipeline (PGAP) v4.11 (11). The genome of strain YW1^T^ was assembled into 322 contigs (*N*_50_, 14,524 bp; *L*_50_, 61; coverage, 1×) for a total size of 2,758,586 bp with a G+C content of 59.1%. A total of 1,944 predicted protein-coding genes were identified, along with 5 rRNAs, 38 tRNAs, and 3 noncoding RNAs.

### Data availability.

Comamonas jiangduensis strain YW1^T^ is available at the CSUR under the reference number Q1714 (= DSM 100319 =  CCTCC AB 2012033  =  KACC 16697). The complete 16S rRNA gene sequence of strain YW1^T^ has been deposited in GenBank under the accession number JQ941713. The genome sequence project (BioProject PRJEB37457) is publicly available under accession number CADEPJ010000000. The raw data have been deposited under SRA accession number ERR4019952 for MiSeq paired-end reads and SRA accession number ERR4020346 for MinION reads.

## References

[1] Davis GHG, Park RWA 1962 A taxonomic study of certain bacteria currently classified as *Vibrio* species. J Gen Microbiol 27:101–119. doi:10.1099/00221287-27-1-101. 13883904

[2] De Vos P, Kersters K, Falsen E, Pot B, Gillis M, Segers P, De Ley J 1985 *Comamonas* Davis and Park 1962 gen. nov., nom. rev. emend., and *Comamonas terrigena* Hugh 1962 sp. nov., nom. rev. Int J Syst Evol Microbiol 35:443–453. doi:10.1099/00207713-35-4-443.

[3] Sun L-N, Zhang J, Chen Q, He J, Li Q-F, Li S-P 2013 *Comamonas jiangduensis* sp. nov., a biosurfactant-producing bacterium isolated from agricultural soil. Int J Syst Evol Microbiol 63:2168–2173. doi:10.1099/ijs.0.045716-0. 23125317

[4] Ravi RK, Walton K, Khosroheidari M 2018 MiSeq: a next generation sequencing platform for genomic analysis. Methods Mol Biol 1706:223–232. doi:10.1007/978-1-4939-7471-9_12. 29423801

[5] Wingett SW, Andrews S 2018 FastQ Screen: a tool for multi-genome mapping and quality control. F1000Res 7:1338. doi:10.12688/f1000research.15931.2. 30254741 PMC6124377

[6] Karamitros T, Magiorkinis G 2018 Multiplexed targeted sequencing for Oxford Nanopore MinION: a detailed library preparation procedure. Methods Mol Biol 1712:43–51. doi:10.1007/978-1-4939-7514-3_4. 29224067

[7] Jain M, Olsen HE, Paten B, Akeson M 2016 The Oxford Nanopore MinION: delivery of Nanopore sequencing to the genomics community. Genome Biol 17:239. doi:10.1186/s13059-016-1122-x. 27887629 PMC5124260

[8] Bankevich A, Nurk S, Antipov D, Gurevich AA, Dvorkin M, Kulikov AS, Lesin VM, Nikolenko SI, Pham S, Prjibelski AD, Pyshkin AV, Sirotkin AV, Vyahhi N, Tesler G, Alekseyev MA, Pevzner PA 2012 SPAdes: a new genome assembly algorithm and its applications to single-cell sequencing. J Comput Biol 19:455–477. doi:10.1089/cmb.2012.0021. 22506599 PMC3342519

[9] Boetzer M, Henkel CV, Jansen HJ, Butler D, Pirovano W 2011 Scaffolding pre-assembled contigs using SSPACE. Bioinformatics 27:578–579. doi:10.1093/bioinformatics/btq683. 21149342

[10] Boetzer M, Pirovano W 2012 Toward almost closed genomes with GapFiller. Genome Biol 13:R56. doi:10.1186/gb-2012-13-6-r56. 22731987 PMC3446322

[11] Tatusova T, DiCuccio M, Badretdin A, Chetvernin V, Nawrocki EP, Zaslavsky L, Lomsadze A, Pruitt KD, Borodovsky M, Ostell J 2016 NCBI Prokaryotic Genome Annotation Pipeline. Nucleic Acids Res 44:6614–6624. doi:10.1093/nar/gkw569. 27342282 PMC5001611

